# Vehicle submersion: an unknown mechanism of road trauma and drowning 

**Published:** 2022-01

**Authors:** Enayatollah Homaie Rad, Ali Davoudi Kiakalayeh

**Affiliations:** ^*a*^ Social Determinants of Health Research Center, Guilan University of Medical Sciences, Rasht, Iran.; ^*b*^ Guilan Road Trauma Research Center, Guilan University of Medical Sciences, Rasht, Iran.

## Abstract

**Background:**

Vehicle submersion is an important cause of death in road traffic injury in Iran. Based on the data of the Iran’s National Registry of Drowning (INRD), a high number of drownings are related to road traffic accidents. In the United States, 11% of overall drownings and about 5% of drowning deaths are related to vehicle submersion. In this review, we tried to find some information about vehicle submersion as an unknown cause of death.

**Methods:**

A review was done using PubMed, Scopus, Web of Science, Google Scholar, SID, and IranMedex. Articles related to vehicle submersion were downloaded and related information was noted.

**Results:**

There are no guidelines on how to rescue from a floated car in Iranian databases. However, there are some guidelines in English which have been revised totally in recent years. However, there are rare pieces of evidence to test the efficacy of these guidelines. The car submersion survival message contains 4 phrases: unbuckle the seatbelts, open or break the car window, remove the children and get out from the floated car. The depth of water is an important issue in car floating and if it is near 40 centimeters, the car will be floated in the water and if the depth is more than 40 centimeters it may turn turtle.

**Conclusions:**

The mechanism of trauma in road vehicle submersion is mixed and might contain both injuries related to the road accident and drowning. New evidences are needed for health policymakers to develop rescue guidelines for escaping submerged vehicles. Evidences can also be used by engineers to make safe cars.

**Keywords:**

Vehicle, Submersion, Road Trauma, Drowning

